# Automatic segmentation of head and neck primary tumors on MRI using a multi-view CNN

**DOI:** 10.1186/s40644-022-00445-7

**Published:** 2022-01-15

**Authors:** Jens P.E. Schouten, Samantha Noteboom, Roland M. Martens, Steven W. Mes, C. René Leemans, Pim de Graaf, Martijn D. Steenwijk

**Affiliations:** 1 Department of Radiology and Nuclear Medicine, Cancer Center Amsterdam, Amsterdam UMC, Vrije Universiteit Amsterdam, De Boelelaan 1117, Amsterdam, The Netherlands; 2 Department of Anatomy and Neurosciences, Amsterdam UMC, Vrije Universiteit Amsterdam, De Boelelaan 1117, Amsterdam, The Netherlands; 3 Department of Otolaryngology – Head and Neck Surgery, Amsterdam UMC, Vrije Universiteit Amsterdam, De Boelelaan 1117, Amsterdam, The Netherlands; 4 De Boelelaan 1108, 1081 HZ Amsterdam, The Netherlands

**Keywords:** Head and neck squamous cell cancer, MRI, Multi-view convolutional neural network, Registration, Segmentation

## Abstract

**Background:**

Accurate segmentation of head and neck squamous cell cancer (HNSCC) is important for radiotherapy treatment planning. Manual segmentation of these tumors is time-consuming and vulnerable to inconsistencies between experts, especially in the complex head and neck region. The aim of this study is to introduce and evaluate an automatic segmentation pipeline for HNSCC using a multi-view CNN (MV-CNN).

**Methods:**

The dataset included 220 patients with primary HNSCC and availability of T1-weighted, STIR and optionally contrast-enhanced T1-weighted MR images together with a manual reference segmentation of the primary tumor by an expert. A T1-weighted standard space of the head and neck region was created to register all MRI sequences to. An MV-CNN was trained with these three MRI sequences and evaluated in terms of volumetric and spatial performance in a cross-validation by measuring intra-class correlation (ICC) and dice similarity score (DSC), respectively.

**Results:**

The average manual segmented primary tumor volume was 11.8±6.70 cm^3^ with a median [IQR] of 13.9 [3.22-15.9] cm^3^. The tumor volume measured by MV-CNN was 22.8±21.1 cm^3^ with a median [IQR] of 16.0 [8.24-31.1] cm^3^. Compared to the manual segmentations, the MV-CNN scored an average ICC of 0.64±0.06 and a DSC of 0.49±0.19. Improved segmentation performance was observed with increasing primary tumor volume: the smallest tumor volume group (<3 cm^3^) scored a DSC of 0.26±0.16 and the largest group (>15 cm^3^) a DSC of 0.63±0.11 (*p*<0.001). The automated segmentation tended to overestimate compared to the manual reference, both around the actual primary tumor and in false positively classified healthy structures and pathologically enlarged lymph nodes.

**Conclusion:**

An automatic segmentation pipeline was evaluated for primary HNSCC on MRI. The MV-CNN produced reasonable segmentation results, especially on large tumors, but overestimation decreased overall performance. In further research, the focus should be on decreasing false positives and make it valuable in treatment planning.

## Background

Head and neck squamous cell cancer (HNSCC) accounts for approximately 3% of cancers world-wide [1]. Head and neck cancer is typically associated with heavy use of alcohol or tobacco, however in recent years the human papillomavirus emerged as a third risk factor in oropharyngeal cancers [2]. Treatment selection is based on the best tradeoff between cure rate and quality of life, and consists of surgery, chemotherapy and radiotherapy or a combination thereof, depending on e.g. the disease stage [3]. Conservative treatment using concurrent chemotherapy and radiotherapy is increasingly applied to patients with advanced stage HNSCC, with locoregional control and organ preservation as the main treatment goals.

Accurate primary tumor delineation is a crucial step in radiotherapy planning and is performed manually or semi-automatically by radiation oncologists [4]. This process is often time consuming and inconsistencies between experts can have significant influence on precision of the treatment [5, 6]. Automatic segmentation of HNSCC using deep learning is currently mostly investigated with computed tomography (CT) [7, 8], fluorodeoxyglucose-positron emission tomography (^18^ F-FDG-PET) or combined PET/CT [9, 10] as an input for delineation of tumors or surrounding organs-at-risk. However, in head and neck cancer, MRI is the preferred imaging modality to detect local tumor extent because of its superior soft-tissue contrast without adverse radiation effects [11]. A few studies with a limited number of patients used single center MRI data that was obtained within a standardized research protocol to automatically segment HNSCC [12, 13]. With dice similarity scores (DSC) between 0.30 and 0.40, their performance is not comparable with the segmentation performance when using PET/CT (DSC above 0.70) and should improve to make it useful for the clinic. Ideally an HNSCC segmentation method produces results independent of anatomical HNSCC location, MRI scanner vendor or acquisition protocol (2D or 3D). Deep learning methods are also being developed constantly to improve performance on medical datasets. Multi-view convolutional neural networks (MV-CNNs) have been successfully used in a variety of segmentation tasks on medical datasets, where three identical networks are trained simultaneously each on a different 2D plane so that information is included from three planes without the computational complexity of 3D patches [14–16].

The aim of this work is to introduce and evaluate a pipeline for automatic segmentation of the primary tumor in HNSCC on MRI with MV-CNN. To achieve this, we developed a registration and segmentation approach that is able to handle patient movement and a variety of anatomical tumor locations within the head and neck region. Segmentation quality was evaluated both in terms of volumetric and spatial performance.

## Materials and methods

### Study population

For this retrospective study, data from two previous studies were combined [17–19]. Cases were included using the following criteria: (1) primary tumor located in the oral cavity, oropharynx or hypopharynx; (2) availability of T1-weighted, short-T1 inversion recovery (STIR) and optionally contrast-enhanced T1-weighted (T1gad) images together with a manual reference segmentation of the primary tumor on the T1-weighted scans before therapy; (3) at least a T2 primary tumor according to the TNM classification (7th -edition) [20]; Following these criteria, we included 220 cases of which the demographical, clinical and radiological details are shown in Table 1. Reference segmentations of primary tumor tissue were constructed manually on the T1-weighted scans within the scope of the previous studies. Experts were allowed to view STIR and optionally the T1gad while delineating tumor. The dataset is not publically available.

**Table 1:** Demographical, clinical and MRI characteristics of the subjects included in this study

Total cases	220
Demographical data	
Age (yrs)	61.9±9.3
Gender	M: 148 (67%)
Tumor locations	
Oral cavity	52 (24%)
Oropharynx	151 (69%)
Hypopharynx	17 (7%)
Tumor classification*	
T2	78 (35%)
T3	45 (21%)
T4	97 (44%)
Lymph node classification**	
N0	78 (35%)
N1	42 (19%)
N2	97 (44%)
N3	3 (1%)
MRI Sequences	
T1	220 (100%)
T1gad	213 (97%)
STIR	220 (100%)
MR vendors	
GE	104 (47%)
Philips	95 (43%)
Siemens	21 (9%)

* Tumor classification was defined according to the TNM criteria (7th edition): In general, T2 = the tumor is between 2 and 4 cm in the greatest dimension; T3 = the tumor is larger than 4 cm in the greatest dimension or invading surrounding structures; T4 = the tumor invades other (critical) tissues. ** Lymph node classification was defined according to the TNM criteria (7th edition): N0 = no regional lymph-node metastases; N1 = metastases to one or more ipsilateral lymph nodes with the greatest dimension smaller than 6 cm; N2 = metastases to contralateral or bilateral lymph nodes with the greatest dimension smaller than 6 cm; N3 = metastases to one or more lymph nodes with the greatest dimension larger than 6 cm. Abbreviations: T1gad = contrast-enhanced T1-weighted; STIR = short-T1 inversion recovery

### MR imaging

Multiple MRI scanners (Siemens, Philips and GE) were used for acquisition of the data used in this work. Protocols differed between these scanners and between the two studies. A 2D STIR (TR/TE/TI=4198-9930/8-134/150-225ms) of the whole head and neck region was performed after which the area of interest was scanned with a 2D T1-weighted (TR/TE=350-800/10-18ms) sequence before and after injection of contrast. In a subset of the data, this area of interest was also scanned with an additional STIR. Due to the use of different scanners and acquisition protocols, reconstruction resolution varied between cases. The (contrast-enhanced) T1-weighted images typically had voxel dimensions of 0.4 × 0.4 × 4.4mm, whereas STIR images had dimensions of 0.4 × 0.4 × 7.7mm.

### Preprocessing

Tools from the FMRIB Software Library (FSL; http://fsl.fmrib.ox.ac.uk) were used to align all subjects and MRI sequences into an isotropic 1-mm head and neck standard space (224 × 192 × 117 pixels), which is common practice in brain lesion studies [21, 22]. Construction of the head and neck standard space was done by merging the T1-weighted images in the dataset as follows. The T1-weighted image of one subject was selected as a global reference and interpolated to 1-mm isotropic space. This selection was done by visual assessment of all images by an experienced radiologist, selecting a case that had a relatively small primary tumor, an average bended neck and the full FOV occupied. The T1-weighted images of all other subjects were first rigidly (FSL FLIRT, default scheme, spline interpolation) and then non-linearly (FSL FNIRT, default scheme, spline interpolation) registered to the global reference in order to form a stack of co-registered T1-weighted head and neck images. The head and neck standard space was then constructed by taking the voxel-wise non-zero average of the stack of T1-weighted images and only including the voxels that were in at least 30% of the registered T1-weighted images.

Then, again, a two-step approach was used to rigidly register (FSL FLIRT, spline interpolation) the T1-weighted image of each participant to head and neck standard space. First, FSL FLIRT was used to obtain an initial affine transformation matrix between subject space and the head and neck standard space. In the second step FSL FLIRT was initialized by the rigid component of the transformation matrix of the first step and weighting was applied to filter out the background. The STIR and T1gad of individual subjects were then also co-registered with the corresponding T1-weighted images using the same two-step approach, and the transformation matrices were concatenated to transform all images to head and neck standard space (spline interpolation).

### Network structure

**Fig. 1:**
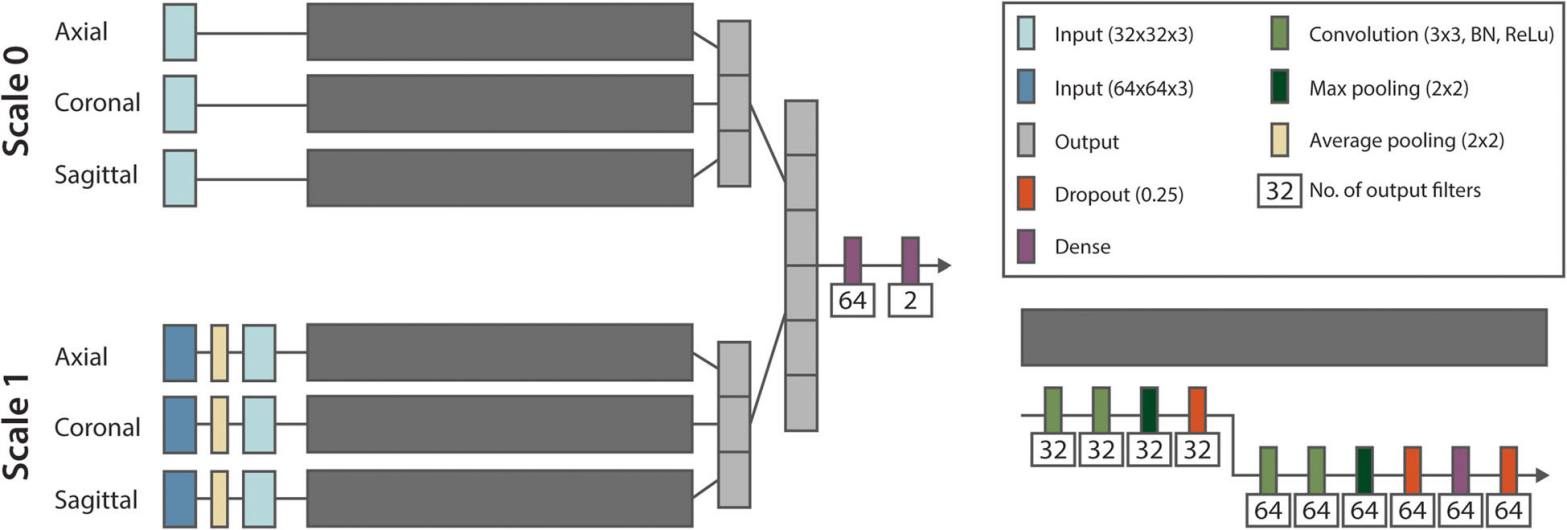
The MV-CNN architecture used in the current study. On the left side, the schematic overview with the pyramid structure (scale 0 and scale 1). Each branch of the MV-CNN has the same structure, which is shown on the right, consisting of convolutional (with batch normalization (BN) and ReLu activation), max pooling, dropout and dense layers. The outputs of the branches are concatenated and with two dense layers reduces to the output of size two, representing non-tumor and tumor

A multi-view convolutional neural network (MV-CNN) architecture was implemented with three equal branches, modelling the axial, coronal and sagittal 2D plane [14]. Together, these branches combine the information of three views with reduced computational complexity compared with 3D patches [23]. A visualization of the network is displayed in Fig. 1. The input of each branch is a 32 × 32 patch with three channels representing the three MRI sequences: T1-weighted, T1gad and STIR. When a sequence was not available in a subject, the channel was zeroed. Each branch consists of a convolutional (3 × 3 kernel, batch normalization, ReLu activation), max pooling (2 × 2 kernel), dropout (25%) and dense layer. The output of each branch is concatenated before passing through two dense layers (ReLu and softmax activation, respectively) to get an output of size two, representing non-tumor and tumor. To include larger-scale contextual information, a pyramid structure was implemented where two inputs (scale 0; 32 × 32 × 3 and scale 1; 64 × 64 × 3) are included for each of the three views [24]. The latter was first downsampled to 32 × 32 × 3 to fit in the network.

### Training procedure

The model was trained on a NVIDIA GeForce GTX 1080 TI graphics processor unit (GPU) with tensorflow-gpu version 1.9.0, CUDA version 10.1 and Python 3.6.7. Because approximately 2% of the MRI image voxels belonged to tumor tissue, we reduced the class imbalance by randomly sampling 50% of all tumor voxels and 1% of all healthy tissue voxels for training. Both the 32 × 32 and 64 × 64 patch were created around the same selected pixel and the channels in each patch were variance normalized to include intensities from four standard deviations from the mean. Only voxels representing healthy of tumor tissue were used for training or testing. To prevent border effects, an extra border of 32 zeros was padded around the full image in all three dimensions. Five-fold cross-validation was used to evaluate performance. Manual quality control ensured the consistency of the distribution of demographical and medical characteristics distributions between the folds. Each model and fold was trained in 25 epochs using a batch size of 512, Adam optimizer [25] and softdice loss function. The softdice loss coefficient was calculated over all voxels within a batch. Using an initial learning rate of 0.001, the learning rate was lowered by 20% after every fifth epoch.

### Evaluation

To obtain full segmentation of the test images, the trained network was applied to all voxels within the mask. After interference the intra-class correlation coefficient (ICC; single measure and absolute agreement [26]) and the Dice Similarity Coefficient (DSC) were calculated to evaluate volumetric and spatial performance. DSC was also compared between subgroups based on tumor classification and location. Because the T-stage in the TNM-classification both includes information on tumor size and invasiveness of the tumor into surrounding tissues, we created four similar sized groups based on tumor volume to compare DSC between these subgroups.

### Statistical analysis

Statistical differences in spatial performance were assessed using Python SciPy package comparing subgroups in tumor classification, location and volume (Wilcoxon rank-sum test). P-values < 0.05 were considered statistically significant.

## Results

**Fig. 2:**
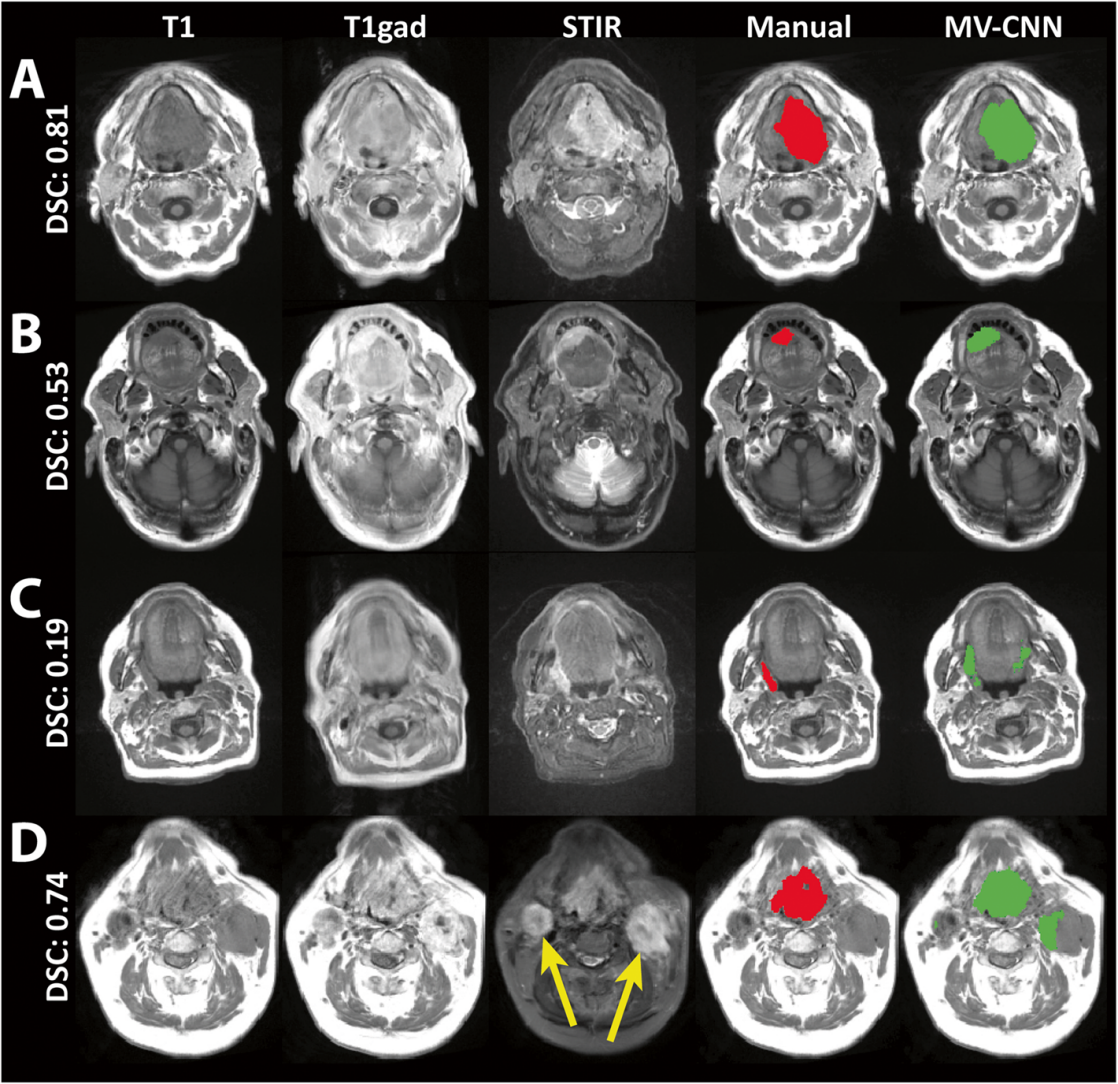
Segmentation results with the three MRI sequences, in red the manual segmentation and in green the network segmentation of the MV-CNN on T1-weighted images. The whole image DSC scores are given per example. On top a good (**A**) and reasonable (**B**) result is shown in an oral cavity (tongue) and floor of the mouth tumor, respectively. False positives in the predicted segmentation were found often. In (**C**) the oropharyngeal (tonsillar fossa) tumor was located on the right side, with false positive classifications on the contralateral side in healthy tissue. In (**D**) a large oropharyngeal cancer (base of tongue) was adequately segmented, however the network also had false positive segmentations in the bilateral lymphadenopathy (yellow arrows)

**Fig. 3:**
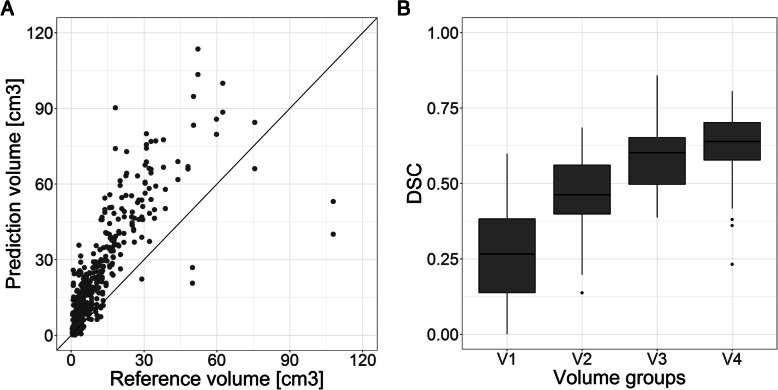
**A** The reference tumor volume plotted against the predicted tumor volume that shows systematic overestimation of the tumor. **B** For the four volume groups, the spatial performance in DSC of the MV-CNN is shown. The spatial performance increases when the tumor volume. V1 = tumor volumes below 3 cm^3^; V2 = tumor volumes between 3 and 7 cm^3^; V3 = tumor volumes between 7 and 15 cm^3^; V4 = tumor volumes above 15 cm^3^

The average reference volume was 11.8±6.70 cm^3^ with a median [IQR] of 13.9 [3.22-15.9] cm^3^. The average MV-CNN tumor volume was 22.8±21.1cm^3^ with a median [IQR] of 16.0 [8.24-31.1]. Average volumetric performance of MV-CNN was ICC=0.64±0.06 and average spatial performance was DSC=0.49±0.19 (Table 2). Figure 2 illustrates four typical segmentation results, of which 2 A and 2B show a good and reasonable result. Figure 2C and 2D illustrate the effect on the spatial performance of the MV-CNN of false positive classifications, both in healthy tissue structures (Fig. 2C) as well as in pathologically enlarged lymph nodes (Fig. 2D). MV-CNN showed a structural overestimation of the predicted tumor volume (Fig. 3A). Although misclassifications of the automatic segmentation often occurred in pathologically enlarged lymph nodes, there was no difference found in model performance between cases from different lymph node subgroups in the TNM classification (Table 2). There is also no difference in the performance between the T3 and T4 subgroups in the TNM classification, only the T2 subgroup scored a lower spatial performance compared with the other subgroups (both *p*<0.001).

**Table 2:** Performance results in ICC and DSC (mean±standard deviation) by the MV-CNN for all test cases and DSCs per subgroup based on tumor classification, location, volume and lymph node classification for the five-fold cross-validation

	N	MV-CNN
INTRA-CLASS CORRELATION (ICC)		
All	220	0.64±0.06
DICE SIMILARITY SCORE (DSC)		
All	220	0.49±0.19
*Tumor classification*		
T2	78	0.39±0.21
T3	45	0.53±0.17
T4	97	0.55±0.15
*Tumor location*		
Oral cavity	52	0.38±0.19
Oropharynx	151	0.51±0.18
Hypopharynx	17	0.57±0.11
*Tumor volume*		
V <= 3 cm^3^	51	0.26±0.16
3 < V <= 7 cm^3^	62	0.47±0.12
7 < V <= 15 cm^3^	50	0.59±0.11
V > 15 cm^3^	57	0.63±0.11
*Lymph node classification*		
N0	78	0.47±0.18
N1	42	0.53±0.20
N2/3	100	0.48±0.19

### Tumor size dependency

Due to the fact that the TNM classification is not only based on tumor size but also on invasiveness in surrounding structures, the spatial performance was measured additionally between four groups of tumor volumes: patients with tumor volume <3 cm^3^ (V1), tumors between 3 and 7 cm^3^ (V2), tumors between 7 and 15 cm^3^ (V3) and tumors >15 cm^3^ (V4). It was found that the spatial performance of the MV-CNN increased significantly with larger tumor volumes (V3 vs. V4 *p*=0.037, all others *p*<0.001), which is also visible in Fig. 3B where these volume groups are plotted against the DSC scores. The smallest tumor volume group scored a DSC of 0.26±0.16 and the largest tumor volume group a DSC of 0.63±0.11.

The oral cavity tumors showed a lower spatial performance than the other two tumor locations (oropharynx and hypopharynx; both *p*<0.001). This could be explained by the lower tumor volumes of included oral cavity tumors (6.4±8.3 cm^3^) in comparison to oropharyngeal (*p*<0.001) and hypopharyngeal (*p*<0.001) tumors (12.9±15.0 and 18.6±13.3 cm^3^, respectively) in our study group.

## Discussion

In this study, we developed and evaluated a pipeline for automatic primary tumor segmentation in HNSCC using three conventional MRI sequences obtained from our multivendor MRI database. The proposed MV-CNN produces segmentations with reasonable volumetric and spatial performances (ICC=0.64±0.06 and DSC=0.49±0.19 respectively).

Only a limited number of studies are available on primary tumor segmentation in the head and neck area solely using MRI data as input. Although the goal of Bielak et al. [12] was different from this study, the segmentation results can be compared to ours. They evaluated the effect of distortion correction in apparent diffusion coefficient (ADC) measurements on the segmentation performance of CNNs. In their study, they used a 3D DeepMedic network structure to segment HNSCC in 18 patients. They found no significant performance difference with or without this distortion correction and received an average DSC of 0.40. They also emphasized the impact of the complexity of the head and neck region and the large variety of sizes, shapes and locations in HNSCC on the performance of an automatic segmentation algorithm. In a more recent study of Bielak et al. [13], only HNSCCs were included with a shortest diameter of at least 2 cm to investigate the segmentation performance. Even though these tumors would classify as larger tumors in our study and full MRI protocols with seven MRI sequences were used as input for the 3D DeepMedic CNN (compared with three sequences in this study), they scored a lower mean DSC of 0.30.

In other previous studies, MRI-based segmentation proved to be able to segment nasopharyngeal carcinoma in the head and neck region with good performance with mean DSCs around 0.80 [27, 28]. However, this type of cancer is not considered to be biologically related to HNSCC [29] and always arises from the nasopharynx epithelium [30], which makes anatomical localization easier than HNSCCs that in our case were located in various anatomical locations within the hypopharynx, oropharynx or oral cavity.

Segmentation of HNSCC with both PET- and CT-scans have been done more frequently in literature and with better results than with MRI. Guo et al. [9] showed a DSC of 0.31 using only CT scans, but reached a DSC of 0.71 when CT was combined with PET images. Other PET/CT studies also show high segmentation performances with DSCs above 0.70 [10, 31–33]. Although the results in this study seem to be an improvement to previous HNSCC segmentation results in MRI, there is still a gap between the performance of MRI and PET/CT that should be overcome first to make MRI as suitable for automatic segmentation of HNSCC as PET/CT. Besides further research in segmentation methods with only MRI input data, the use of data obtained from an integrated PET/MRI system might help in bridging the gap in the future.

The relatively low mean ICC and DSC in this study were mainly driven by false positives, both in the tumor border and in pathologically enlarged lymph nodes. Depending on the tissue within the head and neck region, the transition between tumor and healthy tissue can vary significantly. The registration of the images to the standard space also resulted in loss of data when the voxel size changes to 1mm in all directions, which can also have an effect on the clearness of the border around the tumor. Apart from this, there were also frequently false positively classified structures present in healthy tissues as well as in pathologically enlarged lymph nodes. Lymph node metastases occur frequently in HNSCC and are sometimes even larger in volume compared with the primary tumor. That the network classifies these enlarged nodes as tumor tissue can be understood since the normal anatomy is sometimes significantly distorted due to the local mass effect. It can be hypothesized that an integrated network, which is trained on both primary tumors and lymph node metastases might show a better spatial and volumetric performance compared with networks only based on the primary tumor. Therefore, including manual reference segmentations of the pathologic lymph nodes could potentially further increase the segmentation accuracy of the primary tumor. Another solution might be to manually draw a cube around the tumor in which the network accurately segments the tumor to reduce misclassifications in healthy tissue.

The performance of the segmentation network also depended on size of the primary tumor. Since T-stage in the TNM classification is not based solely on tumor size, but also takes into account tumor invasion into critical anatomical structures, we added the analysis of network performance in categories of tumor volume. Larger tumor volumes showed significantly higher DSCs compared with the low volume tumors. There is only little information on tumor volumes of included HNSCCs used in previously published studies. Besides the fact that smaller tumors are also harder to manually segment for experts, another explanation of this lower spatial performance could be that the DSC of a small object is also more easily affected by false positives because of the smaller true positive area. To be able to really compare performances between studies, information on the included tumor volumes should be provided in published studies.

Head movement (turning or tilting of the head), swallowing and metal artifacts are important causes of MR image artifacts in the head and neck area. Besides hampering the quality of the images, movement will also cause a variable appearance of normal anatomy. To improve network training, we applied registration to the individual MRI images. By first creating a standard space neck by non-linear registration of T1 images, the success rate of the linear registration of each case to this standard space was increased and similarity in the orientation of the scans was created for the network to train with. Linear registration was chosen for the actual registration of the cases, to not alter the anatomical structures in each subject. Because of the linear registration, differences between cases due to swallowing or breathing were not corrected for and could still affect the segmentation performance. The STIR and T1gad scans were also not perfectly aligned to the T1-weighted scan due to the linear registration. The registration could also be improved when patients would lay in a mask during the MRI scan, which is often the case in radiotherapy planning (PET-)CT scans that also have been used for manual segmentation in the cited PET/CT studies [9, 31]. Although the segmentations were evaluated in the standard space in this study, they could be brought back to the original image dimensions of the MRI scans by applying the inverse matrices of the registration process. Further research would be useful to further optimize, or even removing the preprocessing method to make the network more robust for new datasets.

The manual reference segmentation was drawn on the T1-weighted scan, so it is therefore still possible that the manual segmentation did not align perfectly with the other MRI sequences which could also influence the automatic segmentation performance. Improvements can also be made on the manual segmentations of the tumor. Because our study included data from two previous studies, the manual segmentation has been done by two different experts. The exact border between tumor and healthy tissue is sometimes difficult to appreciate in HNSCC, causing a substantial disagreement on the delineation of the tumor between raters [5]. Adding more structural (T2-weighted) and functional (i.e. diffusion weighted imaging and 3D ultrafast dynamic contrast enhanced) MRI sequences could potentially improve manual and automated tumor delineations [34, 35], where it would also be interesting to evaluate the added value of each MRI sequence to the overall performance of a network. Furthermore, the used conventional MRI sequences all consisted of 2D images, which were interpolated to make it usable for our 2.5D approach. The increased availability of functional MRI sequences and 3D MRI sequences for high-resolution diagnostic imaging in HNSCC can further increase automatic tumor segmentation in the future. Because data was used from two previous studies and was obtained by scanners from three different MRI vendors, the data was intrinsically heterogeneous. Although this makes it harder to train the network it can be hypothesized that the result will be more robust and better suited for data acquired in a clinical setting.

## Conclusions

We investigated an automatic segmentation pipeline for primary HNSCC based on MRI data. After registration of the MRI sequences using a head and neck standard space, MV-CNN produced reasonable volumetric and spatial performances, especially in large tumors, but to be able to use the automatic segmentations only on MRI data in treatment planning, the performance has to increase. This could be achieved by reducing the number of false positives in the predicted segmentation.

## Data Availability

The datasets analyzed during the current study are not publicly available.

## Abbreviations

HNSCC: Head and neck squamous cell cancer
CNN: Convolutional neural network
MV-CNN: Multi-view convolutional neural network
ICC: Intra-class correlation
DSC: Dice similarity score
MRI: Magnetic resonance imaging
CT: Computed tomography
PET: Positron emission tomography
STIR: Short-T1 inversion recovery
T1gad: Contrast-enhanced T1-weighted imaging
TR: Repetition time
TE: Echo time
FOV: Field of view
GPU: Graphics processor unit
